# Supplementary material for: Voting intentions during the later stage of the COVID-19 pandemic: The roles of risk perception and performance evaluations in South Korea
Source: PLoS One. 2026 Apr 1;21(4):e0345621. doi: 10.1371/journal.pone.0345621 (PMC13042781; doi:10.1371/journal.pone.0345621)
Supplement: Attachment — Submitted filename: 260105_Response_to_Reviewers.docx [file pone.0345621.s002.docx]

**Response to peer reviewer comments**

Dear Editor:

Thank you for giving us the opportunity to submit a revised version of our manuscript, “**Voting intentions during the later stage of the COVID-19 pandemic: The roles of risk perception and performance evaluations in South Korea” (revised manuscript, PONE-D-25-43702)**, to *PLOS ONE*. We appreciate the time and effort you and the reviewers have dedicated to providing valuable feedback on the manuscript. We are also grateful to the reviewers for their insightful comments on the paper. We have been able to incorporate changes in response to a majority of the suggestions provided by the reviewers. We have included the line numbers of the changes within the response sheet.

Below is a point-by-point response to the reviewers’ comments and concerns.

**Journal Requirement**

1. Please ensure that your manuscript meets PLOS ONE's style requirements, including those for file naming.

**Response:** We have updated the manuscript in accordance with *PLOS ONE*’s style requirements.

2. The journal requires confirmation that the disclosed competing interest affiliation does not affect the authors’ adherence to *PLOS ONE* policies on data and materials sharing.

**Response:** We have updated the competing interest statement according to *PLOS ONE* policies.

3. The journal requires clarification that any restrictions on data sharing are based on legal or ethical grounds, as PLOS ONE permits data to be available upon request only under such conditions.

**Response:** In compliance with *PLOS ONE* policies, the data will be made publicly available at the following address: https://www.openicpsr.org/openicpsr/project/242181/version/V1/view The dataset is currently being prepared for public release and will be accessible immediately after the article is formally published.

**Comments from Reviewer 1**

1. **General**

- I commend the authors clarity of writing and identification of an extremely interesting and timely research question: how long does the rally around the flag (RATF) effect last?

**Response:** Thank you for your positive evaluation of our research.

- The authors convincingly argue that the electoral and political events during the pandemic are an example of RATF, but they were at times followed by a sort of “backlash” or “boomerang” effect afterwards. So, how and why does the RATF effect end?

**Response:** In the revised manuscript, we clarify that our focus is not on the presence or absence of a rally effect per se, but on documenting how different dimensions of risk perception and performance evaluation are associated with voting intentions in the later stage of a prolonged crisis (**Discussion, lines 335–373**).

Specifically, we examine whether affective and cognitive risk perceptions, alongside evaluations of government performance, remained associated with political behavior during the later phase of the COVID-19 pandemic, when acute emotional urgency had diminished and evaluative considerations were more salient **(Introduction, lines 138–160).**

2. **Theory and data collection**

- This work is very interesting and promising. But at this stage, I’m afraid, your empirical and theoretical argument is not sufficiently developed. Your main argument revolves around the distinction between affective and cognitive risk. This is theoretically interesting – but the way you operationalise it is not very convincing. First, has this been done before? If your choice to operationalise as worry v. likely has been adopted before, I think you should say it. If you are introducing a methodological innovation, then you need to be more convincing of its theoretical and empirical validity.

**Response:** Thank you for encouraging us to clarify the theoretical and empirical grounding of our operationalization. In the revised manuscript, we now explicitly situate the distinction between affective and cognitive risk perception within established risk-perception research and clarify that this approach is not intended as a methodological innovation. Specifically, we note that prior research conceptualizes affective and cognitive risk perceptions as interrelated dimensions and that dual-process theory has long distinguished between emotionally and analytically driven modes of risk evaluation. We further clarify that, consistent with existing COVID-19 research, cognitive risk perception is commonly operationalized through perceived likelihood or expected health consequences, while affective risk perception is measured through self-reported worry or emotional concern **(Introduction, lines 77–121)**. These revisions are intended to make clear that our operationalization follows established practice rather than introducing a novel measurement strategy.

- Second, why do you assume that likelihood does not impact worry? I could be more worried about something that is more likely to happen. In your case, however, I am afraid that worry also influences likelihood. If your questions were not randomised, the response on the affective response question might prime respondents on the cognitive questions (people hate to contradict themselves). Hope has been recently theorised as a bi-dimensional concept at the crossroads between valence and likelihood (https://www.researchgate.net/publication/343086814_Hope_During_Conflict). I think you could argue something similar about worry.

**Response:** We appreciate your insightful connection to recent work on hope as a bi-dimensional construct integrating valence and perceived likelihood (Leshem & Halperin, 2020). We neither assume that perceived likelihood does not influence worry nor do we assume unidirectional causality in the opposite direction. Rather, consistent with prior research, we treat affective and cognitive risk perceptions as interrelated components that may mutually influence one another over time, while retaining an analytic distinction for measurement purposes **(Introduction, lines 77–121)**.

We acknowledge your concern regarding potential priming effects arising from question order. However, our aim is not to disentangle causal directionality between worry and likelihood but to examine how each dimension is associated with political attitudes at a specific point in time.

- Finally, in 2022 the pandemic was largely beyond us. Why would people still be worried about it? And likelihood levels might be low, especially depending on vaccination rates (can you say something about that?).

**Response:** While vaccination rates had increased by early 2022, the COVID-19 pandemic was not fully resolved at the time of data collection, as breakthrough infections, emerging variants, uncertainty about long-term health effects, and uneven risk exposure persisted. Accordingly, both affective and cognitive risk perceptions continued to vary meaningfully across individuals, even in the later phase of the pandemic. We have revised the first paragraph of the “Data collection” subsection of the Methodology and added additional citations **(Materials and methods, lines 163–172)**.

- Two other (important) issues: the order of questions should be randomised in survey. And data collection should be pre-registered. If this did not happen, you should tell the reader why and what are the consequences of these choices for the results.

**Response:** The data analyzed in this study were drawn from a nationally representative cross-sectional election survey conducted by Gallup Korea. The questionnaire design, including the order of questions, was finalized prior to data collection and did not allow for subsequent modification. While the order of questions was not randomized, response options within individual questions were randomized by the survey organization, helping to mitigate potential response-order effects. Although question-order effects cannot be entirely ruled out, all respondents were exposed to the same standardized question sequence, which reduces the likelihood of systematic bias in comparisons across groups. To clarify these points, we have revised the text to explicitly describe the survey’s administration by Gallup Korea as part of a nationally representative election survey using computer-assisted telephone interviews **(Materials and methods, lines 174–189)**.

In addition, because the data were not preregistered and were collected as part of a time-sensitive election survey, the analyses are best interpreted as correlational rather than confirmatory. The findings are guided by established theory and consistent with prior research, but they do not support causal claims **(Discussion, lines 383–385)**.

- Finally, you should better engage with literature on dual process theory. Many theoretical and empirical accounts now invite to go beyond this dichotomy. You can disagree but better to acknowledge that the debate exists.

**Response:** We have revised the literature review to more explicitly engage with ongoing debates surrounding dual-process approaches to risk perception. We now clarify that while affective and cognitive risk perceptions are often distinguished in the literature, they are widely understood as interrelated rather than as a strict dichotomy. We further explain why prior research has commonly measured worry and perceived likelihood separately—particularly in contexts such as COVID-19, where protective measures may reduce perceived likelihood without fully alleviating emotional concern—and clarify that the present study follows this established practice. These revisions acknowledge the broader theoretical debate while clarifying our analytic approach **(Introduction, lines 77–121)**.

3. **Results and discussion**

- You should be very clear that you are not making any causal claim. You say “estimates may be biased when interpreted as causal effects”, but this is not accurate. There is no casual effect.

**Response:** We agree that, given the cross-sectional nature of our data, the findings should not be interpreted as causal. In response to your comment, we have revised the manuscript to remove language in the Discussion that could be misinterpreted as implying causality, including the paragraph in question. We hope that this revision clarifies our non-causal analytical stance and reduces any potential confusion for readers.

- The discussion, then, is confusing. You seem to conclude that the most important thing was dissatisfaction with real estate policy, but then why focus on risk perception and covid? In the next paragraph I suggest a way to make sense of this – but it requires more data collection.

**Response:** We agree that the Discussion could be confusing, particularly regarding the emphasis on real estate-specific issues, which risked obscuring the core argument of the paper; accordingly, we have removed these references from the revised manuscript. Instead, we reinterpret the findings by emphasizing that conventional performance evaluations emerged as significant alongside assessments of pandemic response capacity, rather than being displaced by them. Although the cross-sectional design does not allow us to directly examine causal relationships or changes over time, we draw on existing literature on crisis politics and retrospective voting to offer an interpretive account of a transition from pandemic-dominated electoral dynamics to a mixed configuration in which pre-crisis performance considerations re-emerge while pandemic-related issues continue to coexist. To support this reframing, we have substantially revised the Introduction, conceptual framework (Figure 1), and Discussion. We hope these revisions address your concern and clarify the paper’s central contribution **(Introduction, lines 21–160; Discussion, lines 299-380)**.

- Lastly, you make too many assumptions about the mechanisms. From the data you have you can infer very little. You speculate that “affective risk perception may have carried less weight” and “cognitive risk perception may have continued”. These are interesting ideas but you need more supporting evidence to your claims. Maybe you can think of a mixed-methods design and complement survey data with interviews?

**Response:** We agree that the original discussion risked being read as overinterpreting underlying mechanisms that cannot be directly identified with cross-sectional survey data. In the revised manuscript, we have therefore taken care to avoid definitive or mechanistic claims, while retaining an explicitly interpretive and theory-informed discussion of possible explanations that is grounded in and consistent with prior literature. In particular, statements regarding affective risk perception carrying less weight and cognitive risk perception remaining salient are now framed as plausible interpretations rather than empirical demonstrations, informed by existing research on crisis normalization and risk fatigue **(Introduction, lines 77–122; Discussion, lines 303–334)**. At the same time, we explicitly acknowledge that our design does not allow direct examination of mechanisms or temporal change, and we note that mixed-methods approaches, including qualitative interviews, would be necessary to address these questions in future research **(Limitations, lines 383–385)**. We hope these revisions clarify the scope of our claims and directly address concerns about overinterpretation while preserving the analytical value of theory-informed interpretation.

- Minor issue: I wonder if you can adopt a more standard template for the regression table? It’s better if it’s all in one page, and you should have at least another layer of asterisks at p<.01.

**Response:** In line with common practice in published empirical studies, we have merged the summary statistics and odds ratio results into a single table **(Table 1, line 265)** to improve clarity and ensure that the regression results are presented on one page.

Moreover, we have added an additional layer of significance indicators to distinguish between *p* < .01, *p* < .05, and *p* < .10, and we have also included exact p-values in the table to facilitate interpretation and transparency **(see the note below Table 1)**.

4. **Structure and engagement with the literature**

- The first paragraph of the introduction is unnecessary. Try to keep your writing focused on what you are trying to say, cutting down on unnecessary context. To me, there is no harm in starting the article at line 34. You can then immediately introduce South Korea as your case study, but focus more on why it can be a representative case for other countries or broader contexts (external validity). You can take inspiration from recent literature:

Vasilopoulos, P., Marcus, G.E., Valentino, N.A. and Foucault, M. (2019), Fear, Anger, and Voting for the Far Right: Evidence From the November 13, 2015 Paris Terror Attacks. Political Psychology, 40: 679-704. https://doi.org/10.1111/pops.12513

Lytkina, E., & Reeskens, T. (2024). Rally Around the Government or a Populist Response? How Concerns About COVID-19 and Emotional Responses Relate to Institutional Trust and Support for Right-Wing Populism. American Behavioral Scientist, 69(4), 478-504. https://doi.org/10.1177/00027642241240418 (Original work published 2025)

**Response:** Following your comment, we substantially revised and restructured the Introduction to improve focus and theoretical clarity, rather than retaining a broad descriptive overview of the pandemic **(Introduction, lines 21–74)**. The revised manuscript now opens directly with the literature on crisis politics, rally-around-the-flag dynamics, and affective versus cognitive mechanisms. South Korea is introduced early as the empirical case, and the revised Introduction explicitly explains that the country offers a particularly informative analytical setting because nationwide elections were held both during the early and later phases of the COVID-19 pandemic, allowing examination of voting intentions as pandemic-related risks became normalized rather than acute **(Introduction, primarily lines 44–74)**. In addition, the Discussion has been revised to situate the findings within cross-national literature on crisis politics by highlighting how risk perception and retrospective performance evaluations can coexist during the later phase of a prolonged crisis, rather than assuming crisis-driven voting dynamics limited to the acute phase **(lines 335–380)**. We have also incorporated the suggested references into the Introduction and added them to the reference list, using them to inform the revised theoretical framing (e.g., Vasilopoulos et al., 2019; Lytkina & Reeskens, 2024). These revisions clarify the study’s contribution and strengthen its external validity **(Introduction and Discussion, lines 21–74 and 299–402)**.

- In your literature review section, you should be more focussed on what is the argument you are making. Your main RQ is “How does risk perception influence RATF effect after major crises”. Focus on these strands of literature: risk perception/propensity, crises, RATF. There is literature on risk propensity and voting beyond SARF, perhaps have a look whether it’s helpful.

**Response:** We revised the literature review to sharpen its focus on the core argument of the paper—how risk perception relates to rally-around-the-flag dynamics in the context of major crises. The revised section brings together literature on affective and cognitive dimensions of risk perception in political judgment, research on how crises shape and reconfigure these perceptions, and studies on the emergence and attenuation of rally-around-the-flag effects over time **(Literature review, lines 77–144)**. Rather than providing broad contextual background, this section now more directly aligns these strands with the study’s main research question.

- You can use the COVID pandemic as a case study, but I would not make your argument around that. I think you can make it broader to crises in general. Perhaps, since South Korea underwent a major political crisis in 2024/5, you can collect new data and see if and how different crises trigger different RATF effects. And/or you can use the housing crises.

**Response:** Thank you for this helpful suggestion to consider the broader applicability of our findings beyond the COVID-19 pandemic. In the revised manuscript, we frame the analysis to speak to broader dynamics of prolonged crises by focusing on whether risk perception and crisis-related government performance evaluations continue to shape political behavior beyond the most acute phase **(Introduction, lines 21–43**). We also clarify why South Korea offers an analytically informative setting for this question, namely because it experienced two nationwide elections at distinct stages of the same crisis **(Introduction, lines 44–74**). Specifically, the early phase of COVID-19 coincided with the April 2020 National Assembly election, where outcomes were consistent with a pronounced rally-around-the-flag pattern **(Introduction, lines 49–55**), whereas the later stage culminated in the March 2022 presidential election after crisis management had become routinized **(Introduction, lines 56–66**).

In addition, we deliberately avoid linking the 2022 presidential election to subsequent political developments such as the martial law declaration in December 2024, which was widely regarded as unexpected and could not reasonably have been anticipated by voters, thereby preventing post hoc overinterpretation. While examining other crisis contexts would indeed be valuable, we treat such extensions as directions for future research rather than requirements for the present study.

- You also spend too much time on voters evaluation. I think government’s approval is a controlling variable, but does not need to have a central role in your theoretical framework.

**Response:** In the revised manuscript, we clarify that risk perception and retrospective performance evaluations are examined in parallel as analytically distinct but equally important components of the study, corresponding to RQ1 and RQ2, rather than positioning government approval as a mere control variable **(Introduction, lines 145-160)**. Consistent with prior research, we note that conventional retrospective performance evaluations remained salient as the COVID-19 crisis progressed, coexisting with risk-related and pandemic-specific considerations rather than being replaced by them. To address your concern about overemphasis, we have shortened and streamlined the discussion of voter evaluations and clarified their role in the analysis, while providing a more detailed interpretation of retrospective performance **(Literature review, lines 123–144; Discussion, lines 335–380)**.

- One last thing on case selection: Can you tell me if there are any peculiar characteristics of South Korean society which might affect external validity? Any specific social or political traditions, conventions, or cultural worldviews, that may limit your conclusions to SK only? Maybe not, but as a reader I am wondering whether the effects are driven by something else other than risk perception.

**Response:** We understand this comment as questioning whether the observed associations might be driven by characteristics specific to South Korean society, rather than by risk perception itself. In the revised manuscript, we clarify that South Korea is not treated as a culturally exceptional case but as an analytically informative setting selected because nationwide elections occurred at different stages of the same prolonged crisis, enabling examination of how risk perception and retrospective performance evaluations operate as crises become normalized **(Introduction, lines 44–74)**.

We acknowledge that contextual features such as relatively high state capacity and institutionalized crisis-response practices may condition the timing or magnitude of political responses to crises (**Discussion, lines 349–380**). However, these factors are treated as conditioning contexts rather than alternative explanations that displace risk perception as the central mechanism of interest. Accordingly, we emphasize that the findings should be interpreted in light of South Korea’s institutional context—not because it is culturally exceptional but because it provides a particularly informative setting for examining how electoral behavior unfolds during a prolonged crisis—while leaving open the possibility that similar dynamics may be examined in other contexts in future research **(Limitations, lines 388–390)**.

**Comments from Reviewer 2**

- A conceptual model figure summarizing hypothesized relationships among variables would enhance reader understanding.

**Response:** We have now added Figure 1, which presents a conceptual framework summarizing the key relationships examined in the study. The figure illustrates how political responses are situated across different phases of a prolonged crisis, highlighting the transition from rally-oriented dynamics during the acute phase to the re-emergence of pre-crisis political considerations as crisis management becomes routinized. It also clarifies how risk perception and retrospective performance evaluations correspond to RQ1 and RQ2, respectively, and how both are examined within a shared empirical context using data from a single cross-sectional survey. We hope this figure improves the clarity of the theoretical structure and helps readers better understand the scope and positioning of the analysis.

Figure 1. Conceptual framework of crisis phases and political responses.

Note: RQ1 = Research question 1; RQ2 = Research question 2.

- Clarify in the methods section whether the weighting was applied in regression analysis or only for descriptive statistics.

**Response:** We would like to clarify that weighting was applied to both the regression analysis and chi-squared tests based on descriptive statistics, but the descriptive statistics were not weighted statistics. We have updated the table to reduce confusion. We note that the overall patterns remain consistent despite minor changes at the decimal level. We have added additional explanations to clearly explain these changes. **(Materials and methods, lines 174–189, 237-239; Table 1, line 265)**.

- Improve phrasing in the abstract’s conclusion—currently, the statement that “performance voting may outweigh the rally-around-the-flag effect” may overstate the evidence; softening this claim would improve precision.

**Response:** We agree that the original wording overstated the evidence, and we have therefore substantially revised the abstract, including its concluding sentence, to improve precision. The revised conclusion now states that voting intentions were associated with conventional performance evaluations alongside pandemic-response approval, without implying that one mechanism outweighed another **(Abstract, lines 2–16)**.

**Comments from Reviewer 3**

- The manuscript was written very clearly in English, which made it easy to follow. It presented significant and important new findings that were well-supported because all the data was transparent and simple to see. We could also easily understand the statistical analysis, making the whole paper exceptionally clear and its conclusions highly reliable..

**Response:** Thank you for your generous and encouraging evaluation of our manuscript. We are grateful that you found the paper clear, the analysis transparent, and the conclusions reliable. Your positive feedback is appreciated and encouraging for our work.
